# Pattern Electroretinogram Parameters Are Associated with Optic Nerve Morphology in Preperimetric Glaucoma after Adjusting for Disc Area

**DOI:** 10.1155/2021/8025337

**Published:** 2021-10-13

**Authors:** Andrew Tirsi, Vasiliki Gliagias, Julie Moehringer, Derek Orshan, Sofia Tello, Peter Derr, Sung Chul Park, Stephen A Obstbaum, Celso Tello

**Affiliations:** ^1^Manhattan Eye Ear and Throat Hospital, New York, NY 10065, USA; ^2^Donald and Barbara Zucker School of Medicine at Hofstra University/Northwell Health, Hempstead, NY 11549, USA; ^3^Sandford H. Calhoun High School, Merrick, NY 11566, USA; ^4^New York Institute of Technology College of Osteopathic Medicine, Old Westbury, NY 11545, USA; ^5^Rye High School, Rye, NY 10580, USA; ^6^Diopsys Inc., Pine Brook, NJ 07058, USA

## Abstract

**Purpose:**

We examined the relationships between pattern electroretinogram and optical coherence tomography derived optic nerve head measurements, after controlling for disc area.

**Methods:**

Thirty-two eyes from 20 subjects with preperimetric glaucoma underwent pattern electroretinogram and optical coherence tomography. Pattern electroretinogram parameters (Magnitude, MagnitudeD, and MagnitudeD/Magnitude ratio) and optic nerve head measurements (rim area, average cup to disc ratio, vertical cup to disc ratio, cup volume, retinal nerve fiber layer thickness sectors, and Bruch's membrane opening-minimum rim width thickness sectors) were analyzed after controlling for disc area.

**Results:**

Magnitude and MagnitudeD were significantly associated with rim area (*r* *≥* 0.503, *p* ≤ 0.004). All pattern electroretinogram parameters significantly correlated with Bruch's membrane opening-minimum rim width sectors—temporal superior and nasal inferior (*r* *=* 0.400, *p*=0.039)—and retinal nerve fiber layer sectors—superior, nasal superior, and inferior (*r* ≥ 0.428, *p* ≤ 0.026). Magnitude and MagnitudeD explained an additional 26.8% and 25.2% of variance in rim area (*B* *=* 0.174 (95% CI: 0.065, 0.283), *p*=0.003, and *B* *=* 0.160 (95% CI: 0.056, 0.265), *p*=0.004), respectively. MagnitudeD and MagnitudeD/Magnitude ratio explained an additional 13.4% and 12.8% of the variance in Bruch's membrane opening-minimum rim width global (*B* *=* 38.921 [95% CI: 3.872, 73.970], *p*=0.031, and *B* *=* 129.024 (95% CI: 9.589, 248.460), *p*=0.035), respectively. All Bruch's membrane opening-minimum rim width sectors and retinal nerve fiber layer sectors (nasal superior, nasal inferior, and inferior) were significantly correlated with rim area (*r* *≥* 0.389, *p* ≤ 0.045).

**Conclusion:**

PERG abnormalities can predict rim area loss in preperimetric glaucoma after controlling for disc area. We recommend controlling for disc area to increase diagnostic accuracy in early glaucoma.

## 1. Introduction

Glaucoma, a disease of progressive optic neuropathy, features optic disc cupping and consequent visual field (VF) deficits [1]. Due to the optic nerve's composition of retinal ganglion cell (RGC) axons, any damage to the optic nerve results in changes to the ganglion cell layer, retinal nerve fiber layer (RNFL), intrapapillary region of the optic nerve head (ONH), and ONH morphology [2]. Disease typically begins as subclinical with a prolonged occult period characterized by normal perimetric fields [3]. While these preperimetric glaucoma (PPG) patients have no symptoms, progressive disc cupping and RNFL thinning may already be present [4–8].

Structural change in the optic nerve precedes VF change, [9] and at least 25% of RGCs must be lost to detect a significant change on perimetry [4]. Sensitive technologies can quantify structural nerve changes to better monitor progression. Studies have shown that optical coherence tomography (OCT) may detect significant loss of the RNFL several years before development of VF loss [10]. Pattern electroretinogram (PERG), which measures retinal response to a contrast reversing pattern, provides information about central macular RGC function [11]. Even in the presence of a normal RNFL thickness and VF, PERG abnormalities found in eyes with ocular hypertension (OHT) or glaucoma suspects can suggest early functional damage [12]. In the work of Ventura et al., PERG was abnormal in amplitude, phase, or interocular asymmetry in amplitude and phase in 52% of glaucoma suspects and 69% of early manifest glaucoma patients, confirming the high sensitivity of PERG for glaucoma detection [13]. Bach et al. studied PERG in OHT patients, confirming it can help predict stability or progression to glaucoma at least 1 year ahead of conversion [14].

A morphologic target in diagnosis of glaucoma, the neuroretinal rim (NRR) area is an intrapapillary reflection of the amount of optic nerve fibers [15]. The correlation between NRR rim area and disc area holds importance in evaluation of glaucoma: the larger the disc, the larger the rim [2]. If only total rim area is taken as criterion for disease, healthy but small optic discs with a small rim area will be classified as glaucomatous, with the opposite occurring in large optic discs [2]. Disc area has shown correlation with cup area and cup volume as well. Thus, similar conclusions can be drawn when considering a high cup to disc (C/D) ratio in a physiologically large optic disc; the high C/D ratio would be considered abnormal despite normal optic nerve morphology [2]. Finally, disc area and Bruch's membrane opening-minimum rim width (BMO-MRW) have a relationship in which small discs may have thicker BMO-MRW than regular-sized discs and a weak correlation with RNFL thickness [16]. Disc size may lead to discrepancy between an abnormal RNFL thickness and normal BMO-MRW [16].

Following the above discussion on the importance of ONH morphology in glaucoma diagnosis and the impact of taking disc size into consideration when assessing ONH morphology, we aimed to examine the relationships between PERG parameters and ONH morphologic measures, after accounting for disc area variability in PPG participants.

## 2. Materials and Methods

Twenty PPG subjects (32 untreated eyes) with normal Humphrey 24-2 VF tests and suspicious ONH were consecutively recruited at Manhattan Eye, Ear and Throat Hospital. In this cross-sectional study, participants underwent a complete comprehensive ophthalmologic examination, PERG tests using Diopsys^®^ NOVA PERG (Diopsys^®^, Inc. Pine Brook, NJ), and OCT testing using Cirrus (Carl Zeiss Meditec, Inc. Dublin, CA) and SPECTRALIS® OCT (Heidelberg Engineering, Inc., Heidelberg, Germany). In this study, only scans with a signal strength greater than 7 were used, as per Cirrus HD-OCT recommendations (signal strength ≥6) [17]. OCT scan quality was assessed by glaucoma specialists (CT, SP) for segmentation errors and artifacts, and no scans with such errors were found in this study. The study was approved by the Institutional Review Board of Northwell Health System. Written informed consent was obtained from all subjects, and the study adhered to the tenets of the Declaration of Helsinki.

PPG participants were recruited according to the following criteria: the presence of a glaucomatous ONH appearance (C/D ratio asymmetry of >0.2 between fellow eyes, NRR thinning, notching, or excavation) and a normal Humphrey Field Analyzer (HFA) 24-2 Swedish Interactive Thresholding Algorithm (SITA) standard test at the baseline visit. Participants within 18–80 years of age with best corrected visual acuity better than or equal to 20/40, spherical refraction within ±6.0 *D*, and cylinder correction within 3.0 *D* were included. Using HFA 24-2 SITA-standard test, only participants with stage 0 (no VF losses) based on the Glaucoma Staging System (GSS 2) were enrolled in this study [18]. A normal HFA test was defined by a Glaucoma Hemifield Test (GHT) within normal limits, pattern standard deviation (PSD) within 95% confidence limits, and mean deviation (MD) ≥ −2 dB. Individuals with unreliable HFA results with fixation losses, false positive rate, or false negative rate >20% were excluded. Participants with prior intraocular surgery except uncomplicated cataract extraction, ocular trauma, or ocular or systemic conditions that may affect the ONH or retinal structure or function were excluded. No participants received intraocular pressure (IOP) lowering treatment at the time of enrollment. OCT images with low quality, visible eye motion, blinking artifacts, or algorithm segmentation failures were considered of poor quality and discarded.

### 2.1. Pattern Electroretinography Testing

The steady state (ss-PERG) was recorded using a commercially available system, Diopsys^®^ NOVA-ss-PERG Contrast Sensitivity module. The test was performed in a dark room to standardize environment luminance—free of visual and audible distractions. The patient's seat height was adjusted so the tested eye stayed in a horizontal plane with the center of the monitor. The forehead skin was cleaned using NuPerp® Skin Prep Gel (Weaver and Company, CO, USA) and the lower eyelids, using OCuSOFT® Lid Scrub Original (OCuSOFT® Inc., Rosenberg, TX, USA) to ensure good and stable electrical activity. Disposable hypoallergenic skin sensors Silver/Silver Chloride ink (Diopsys® proprietary skin sensor) were applied on the lower lids of both eyes, close to the lid margins while avoiding eyelashes. One ground sensor (Diopsys® EEG electrode) was applied in the central forehead area with a small amount of conductive paste (Ten20®, Weaver and Company). Then, cables from the Diopsys® NOVA ss-PERG device were connected to the electrodes. A total of 3 electrodes were used per test per patient (two active/reference and one ground electrodes). Subjects were fitted with the appropriate correction for a viewing distance of 24 inches and were instructed to fixate on a target at the center of the monitor in front of them. An occluding lens was inserted into the trial lens to cover the eye that was not being tested. No pupil dilation was needed. Subjects were asked to blink freely. However, if more than 4 artifacts were recorded over one 25-second test period, subjects were subsequently instructed to reduce blinking frequency and eye lubricants were offered when needed.

The stimulus was presented on a gamma corrected Acer V176BM 17-inch monitor, having a refresh rate of 75 frames/second. Luminance output over time was verified using a luminance meter MAVO-SPOT 2 USB (Gossen, GmbH, Nuremberg; Germany). The pattern stimulus consisted of black/white alternating square bars, reversing at 15 reversals/second (rps) with a duration of 25 seconds for high contrast (HC 85%) and 25 seconds for low contrast (LC 75%) for a total of 50 seconds per eye. The stimulus field subtends a visual angle of 1439.90 arc minutes. Each bar will subtend 22.49 arc minutes, for a total of 64 bars. A red target subtending 50.79 arc minutes was used as a fixation target and was centered on the stimulus field. The luminance of the white bars for 85% and 75% contrast was 204 cd/m^2^ and the luminance for black was 20.5 cd/m^2^ and 52.5 cd/m^2^ yielding a mean luminance of 112.3 cd/m^2^ and 128.2 cd/m^2^, respectively. All recorded signals underwent band filtration (0.5–100 Hz) and amplification (gain = 20,000) and averaged at least 150 frames. The signal was sampled at 1920 samples per second by an analog to digital (A/D) converter. The voltage range of the (A/D) converter was programmed between −5 V and +5 V. Sweeps contaminated by eye blinks or gross eye saccades were rejected automatically over a threshold voltage of 50 *μ*V, and these sections were identified as “artifacts” in the report. Synchronized single-channel electroretinograms (ERGs) were recorded, generating a time series of 384 data points per analysis frame (200 ms). An automatic fast Fourier transformation (FFT) was applied to the PERG waveforms to isolate the desired component at 15 rps. Other frequencies, such as those originating from eye muscles, were rejected. The ss-PERG test results were saved in a Structured Query Language (SQL) database and presented in a report form to be used for analysis. For every subject, four preprogrammed full “contrast sensitivity protocols” were performed one after another. A “contrast sensitivity protocol” consisted of two 25-second recordings for each eye: first with high contrast (85%) diffuse retinal stimulation and then with low contrast (75%) pattern stimulation. The device collects 5 frames of data per second, totaling 125 frames of data, and the first 10 frames (2 seconds) of data are discarded. A result was categorized as nonreliable if there were more than 4 artifacts.

For each eye, three PERG measurements (Magnitude (Mag), MagnitudeD (MagD), and MagD/Mag ratio) were collected and calculated, as well as the number of artifacts and signal to noise ratio (SNR). Mag (*µ*V) represents the amplitude of the signal strength at the specific reversal rate of 15 Hz in the frequency domain, while MagD (*µ*V) represents an adjusted amplitude of the PERG signal impacted by phase variability throughout the waveform recording. A recording where the phase of the response is consistent will produce a MagD value close to that of Mag, whereas a recording where the phase of the response varies will produce a MagD value lower than that of Mag. This is due to the fact that averaging responses which are out-of-phase with each other will cause some degree of cancellation. The MagD/Mag ratio is a ratio that is a within-subject representation of the phase consistency of ss-PERG. The SNR represents the level of electrical noise compared with the level of the PERG signal at 15 Hz.

### 2.2. OCT-Based ONH Morphology Measurements

The Glaucoma Module Premium Edition (GMPE) software was used for the SPECTRALIS® Spectral Domain (SD) OCT (Heidelberg, Engineering, Inc., Heidelberg, Germany). It measures the minimum distance between the internal limiting membrane and Bruch's membrane opening around the optic nerve head, also known as BMO-MRW, which increases accuracy of the anatomic disc margin and the NRR measurements. This software uses the center of the BMO-MRW and of the fovea as fixed landmarks in creation of an anatomic map of the patient's eye, allowing higher sensitivity for structural change [19]. Cirrus® High Definition (HD) OCT (software version 9.0.0.28) was used in this study to provide ONH measurements as described elsewhere [20, 21].

### 2.3. Statistical Analyses

Descriptive statistics were used to evaluate continuous and demographic data. Mean and standard deviations values were determined for each ss-PERG (Mag, MagD, and MagD/Mag ratio), HFA SITA-Standard (24-2) tests, and all ONH OCT-based measurements.

A Pearson correlation analysis was conducted between PERG parameters (Mag, MagD, and MagD/Mag ratio) and ONH measurements (rim area, disc area, average C/D ratio, vertical C/D ratio, cup volume, and BMO-MRW sectors). Partial correlation analysis was conducted after controlling for disc area. In the prediction of the rim area change, a hierarchical linear regression was used, where the disc area was entered in step 1 of the model, and Mag was entered in step 2. An identical model was used by replacing Mag with MagD and subsequently with MagD/Mag ratio in step 2.

In the prediction of BMO-MRW global, an identical hierarchical model was used, after controlling for disc area (step 1). PERG parameters were entered one by one in step 2.

Statistical analyses were performed with commercially available software (IBM® SPSS® ver.23.0; SPSS Inc, Chicago, IL, USA).

## 3. Results

Thirty-two eyes (20 patients) with PPG were initially recruited. All 32 eyes had testing with PERG and Cirrus OCT. 28 eyes additionally had testing with SPECTRALIS® OCT (BMO-MRW, RNFL sectors). The characteristics of the study population are summarized in Tables 1 and 2. Mean age was 58.57 years, and 13 participants were females (65%). The baseline mean HFA MD 24-2 was 0.25 dB and mean IOP was 17.73 mmHg.

### 3.1. Relationships between PERG Parameters and Rim Area

Pearson analysis showed a significant correlation between PERG parameters (Mag and MagD) and disc area (*r* ≥ 0.508, *p*=0.003), rim area (*r* ≥ 0.458, *p* ≤ 0.008), and average C/D ratio (*r* ≥ 0.353, *p* ≤ 0.047). No significant correlations were found between PERG parameters and vertical C/D ratio as well as cup volume. After controlling for disc area, Mag and MagD remained significantly correlated with rim area (*r* ≥ 0.503, *p* ≤ 0.004). No significant correlation was found between PERG parameters and average C/D ratio, vertical C/D ratio, and cup volume (Table 3). In two separate hierarchal linear regression models, in the prediction of rim area, after controlling for disc area (step 1), Mag (step 2) explained an additional 26.8% of the variance in rim area (*B* *=* 0.174 (95% CI: 0.065, 0.283), *p*=0.003). When Mag was replaced by MagD in the same model, MagD (step 2) explained an additional 25.2% of variance in rim area (*B* *=* 0.160 (95% CI: 0.056, 0.265), *p*=0.004) (Table 4). MagD/Mag ratio did not explain any variance in rim area.

### 3.2. Relationships between PERG Parameters and BMO-MRW

After controlling for disc area, all PERG parameters were significantly correlated with BMO-MRW sectors nasal inferior (NI) (*r* ≥ 0.431, *p* ≤ 0.025) and temporal superior (TS) (*r* ≥ 0.400, *p* ≤ 0.039). Mag and MagD were significantly correlated with the temporal (*T*) sector (*r* ≥ 0.411, *p* ≤ 0.033). MagD was significantly correlated with BMO-MRW global (*r* *=* 0.416, *p*=0.031), NI (*r* *=* 0.545, *p*=0.003), *T* (*r* *=* 0.411, *p*=0.033), and TS (*r* *=* 0.433, *p*=0.024) sectors. MagD/Mag ratio was significantly correlated with BMO-MRW global (*r* *=* 0.407, *p*=0.035), NI (*r* *=* 0.431, *p*=0.025), nasal superior (NS) sectors (*r* *=* 0.459, *p*=0.016), and TS (*r* *=* 0.408, *p*=0.034) (Table 5). In the prediction of BMO-MRW global, an identical linear hierarchical regression model was used. After controlling for disc area (Step 1), MagD explained 13.4% of the variance (*B* *=* 38.921 (95% CI: 3.872, 73.970), *p*=0.031). After replacing MagD by MagD/Mag ratio, the ratio (step 2) explained an additional 12.8% of variance (*B* *=* 129.024 (95% CI: 9.589, 248.460), *p*=0.035) (Table 6).

### 3.3. Relationships between PERG Parameters and RNFL

Pearson analysis showed that all PERG parameters were significantly associated with RNFL sectors global, inferior (I), superior (S), nasal (N), and NS (*r* *≥* 0.408, *p* ≤ 0.031). Mag and MagD were significantly associated with RNFL sectors NI and TS (*r* *≥* 0.432, *p* ≤ 0.022). MagD and MagD/Mag ratio were significantly associated with RNFL sector temporal inferior (TI) (*r* ≥ 0.409, *p* ≤ 0.031). After controlling for disc area, all PERG parameters remained significant with RNFL sectors I, S, and NS (*r* ≥ 0.428, *p* ≤ 0.026). Mag and MagD remained significantly correlated with NI and were correlated with global (*r* ≥ 0.422, *p* ≤ 0.028). Only MagD/Mag ratio remained significantly associated with N (*r* *=* 0.422, *p*=0.028) (Table 7).

### 3.4. Relationship between Rim Area, BMO-MRW, and RNFL Thickness Measurements

After controlling for disc area, rim area was significantly correlated with all BMO-MRW sectors (*r* *≥* 0.497, *p* ≤ 0.008) (Table 8) and with RNFL sectors I, NI, and NS (*r* *≥* 0.389, *p* ≤ 0.045) (Table 9).

### 3.5. Scatter Plots Analysis after Controlling for Disc Area

**Figure 1:**
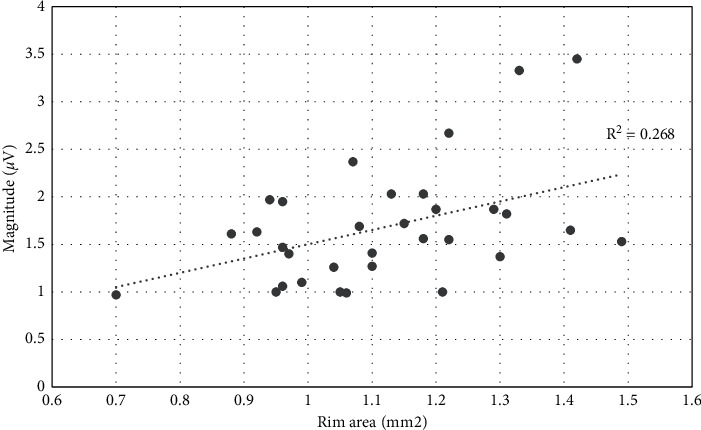
Scatter plot of the relationship between Magnitude and rim area after adjusting for disc area (*r*^2^ = 0.268; *p*=0.003).

**Figure 2:**
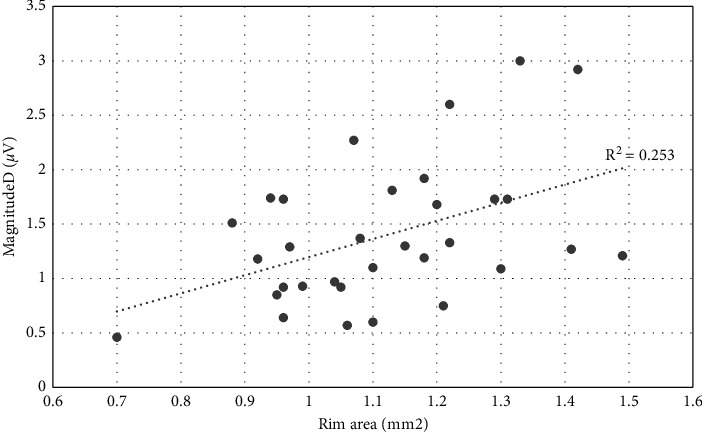
Scatter plot of the relationship between MagnitudeD and rim area after adjusting for disc area (*r*^2^ = 0.253; *p*=0.004.

**Figure 3:**
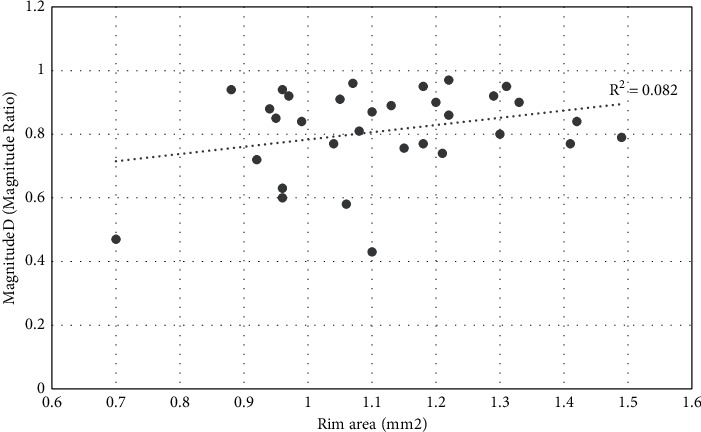
Scatter plot of the relationship between MagnitudeD/Magnitude ratio and rim area after adjusting for disc area (*r*^2^ = 0.082; *p*=0.119).

Scatter plot analysis was used among PERG parameters and rim area, after controlling for disc area. Results have shown significant relationships for Mag (*R*^*2*^ *=* 0.268, *p*=0.003) (Figure 1) and for MagD (*R*^*2*^ *=* 0.253, *p*=0.004) (Figure 2). No significant relationship was found between MagD/Mag ratio and rim area (*R*^*2*^ *=* 0.082, *p*=0.119) (Figure 3).

## 4. Discussion

### 4.1. Rationale of Adjusting for Disc Size

Assessment of RGC damage is necessary in management of glaucoma. Measures like perimetry and OCT allow for clinical decision-making, but we have yet to establish a diagnostic tool which allows for the quantification of remaining or lost RGCs and their axons [22, 23]. The NRR is one of the main parameters used in the diagnosis of glaucomatous optic neuropathy, and NRR is mostly defined by its area (mm^2^) besides its shape and pallor [24].

The optic disc itself is a representation of nerve fiber health as the size of the optic disc is positively correlated to the number of nerve fibers present [2, 25–27]. Size of the optic disc varies widely—about 1 : 7 in a normal Caucasian population—and larger disc sizes exist among black individuals [2, 21, 28, 29]. Eyes with large optic discs compared to eyes with small optic discs have a larger NRR area and a larger number and total area of lamina cribrosa pores [2]. The importance of considering disc size in evaluation of glaucoma inspired our decision to control for disc area when analyzing PERG parameters and ONH morphology measures. Despite recent advancements in OCT technology, studies have been inconclusive concerning the accuracy of disc area measurement [30, 31]. In this study, PERG parameters, Mag and MagD, showed a significant correlation with disc area, suggesting that as disc area decreases, so does the number of retinal nerve fibers which leads to reduction in PERG (amplitude) parameters. Furthermore, after controlling for disc area, the correlation between PERG parameters and rim area improved (Table 3).

In the current study, our measurements were taken using both SPECTRALIS® and Cirrus OCT devices. Mwanza et al. showed that Cirrus HD-OCT ONH parameters, particularly vertical rim thickness, rim area, and vertical C/D ratio, have excellent ability to discriminate between normal eyes and those with even mild glaucoma [21]. Thus, we used Cirrus OCT for the ONH morphology parameters such as disc area, rim area, average C/D ratio, vertical C/D ratio, and cup volume. Cirrus SD-OCT identifies termination of Bruch's membrane as the edge of the disc, which helps in obtaining more consistent and clinically accurate measurements [20]. However, even this technology had limitations. Moghimi et al. examined the disc and rim areas in healthy and glaucomatous subjects, measured with Heidelberg Retinal Tomography and Cirrus SD-OCT [31]. While they did not find a significant change in average disc size when correcting Cirrus measurements for eye magnification, myopic eyes with differing amounts of disc tilt can have premature endings of Bruch's membrane before what is perceived to be the clinical border of the disc on the temporal side [31]. In this study, we acknowledge that optic disc size is almost independent of the refractive error of the eye within a range of −5 to +5 Diopters [2].

In addition to wide optic disc variability and myopia disrupting disc area measurement, further factors contribute to underestimation or overestimation of this variable. While SD-OCT devices like Cirrus OCT are known to have advancements such as enhanced resolution, reduced acquisition time, and less operator dependence, limitations exist which can affect accuracy of measurements such as disc area [30]. For example, although this device allows automated delineation of the optic disc and cup margins, floaters and/or peripapillary atrophy can lead to overestimation of disc area, while blood vessels and motion artifacts can lead to underestimation [30]. Further investigation is needed to definitively state that Cirrus OCT can accurately measure disc area and prevent this variable from confounding other measurements.

### 4.2. Associations between PERG and Rim Area after Controlling for Disc Area

For at least two decades, PERG has been known to detect RGC dysfunction in patients with OHT and normal VF or glaucoma suspects [32–35]. Studies have found that PERG amplitude and peak time represent RGC count, with amplitude reduction suggesting a sign of lost RGCs, dysfunctional RGCs, or both [36, 37]. Banitt et al. showed that, in glaucoma suspects, PERG signal can detect an equivalent loss of OCT signal by several years, and there is an 8-year time lag between PERG amplitude and RNFL thickness to lose 10% of their initial values [38]. In the work of Jeon et al., glaucoma suspects were shown to have correlations between PERG amplitude with disc morphology and RNFL thickness [39]. In particular, the cup morphology measures showed meaningful relationships with PERG amplitudes, agreeing with the degenerative pattern of morphological change to the optic disc first, subsequently ganglion cell dysfunction and/or death, and then sustained mechanical stress for the structural change to the axon [39].

Most of these studies involved transient state PERG (ts-PERG) devices with reversal rate of the checkerboard pattern visual stimulus <15 reversals/second (low temporal frequencies). The ss-PERG differs from ts-PERG in that it operates at higher temporal frequencies, above 10 reversals/s, causing overlap of the successive waveforms. Mag (amplitude) reflects the strength of the electrical response and corresponds to the number of living RGCs. MagD (latency) indicates the presence of RGCs in distress, and it can be thought of as the timing of the RGC response. MagD/Mag ratio is the ratio between the two, and the closer MagD values are to Mag values, the better the RGC function is. In this study, we used ss-PERG technology with the reversal frequency of 15 reversals/second, providing better diagnostic capabilities in early glaucoma [40, 41]. ss-PERG has been considered to provide a higher amplitude, improved latency response, and improved sensitivity over ts-PERG [42–45]. This phenomenon has been explained by RGCs being submitted to a greater metabolic stress during ss-PERG [46–48].

In this study, after controlling for disc size, Mag and MagD were significantly correlated with rim area (Table 3), suggesting the presence of either RGC loss, dysfunctional RGCs, or a combination of both conditions simultaneously. Linear regression models showed that, in the prediction of rim area, after controlling for disc area, Mag explained an additional 26.8% of the variance in rim area. MagD explained an additional 25.2% of variance in rim area (Table 4). Furthermore, all PERG parameters were significantly correlated with most RNFL sectors, and the association increased after controlling for disc size. These findings suggest that the more dysfunctional the RGCs, the smaller the rim area and the thinner the RNFL thickness [15, 49]. The earliest signs of RGC axonal damage include synaptic loss which leads to thinning of the proximal and distal dendrites, abrupt reductions in dendritic process diameter at branch points, and a general decrease in the complexity of the dendritic tree; this leads to reduction in axonal thickness and shrinkage of soma size [50–53]. Thus, our results propose a degenerative pattern in which morphologic change occurs to the disc with possible concurrent RGC dysfunction and subsequent axonal damage, accompanied by delay of axonal transport. Therefore, MagD represents phase delays and an opportunity to detect RGC dysfunction preceding cell death [34]. At this stage, RGC damage is potentially reversible [35, 54].

### 4.3. Relationships between PERG and BMO-MRW after Controlling for Disc Area

Bruch's membrane opening-minimum rim width consists of the minimum distance between the BMO, considered to be the outer border of the neural tissues at the optic nerve head, and the internal limiting membrane [16, 55–60]. It has been reported as a more accurate reflection of the amount of neural tissue from the optic nerve [16]. NRR is lost in all sectors in glaucoma. However, I and TS disc regions have greater involvement in modest glaucomatous damage [2]. Application of the ISNT rule assessed by BMO-MRW has been shown to have better performance in distinguishing healthy from glaucomatous optic discs than when using disc photographs [61]. In the present study, all PERG parameters and BMO-MRW were found to have significant relationships after controlling for disc area (Table 5). The regression analyses had shown that MagD and MagD/Mag ratio were significant predictors of BMO-MRW global, after controlling for disc area (Table 6). These findings suggest that RGC dysfunction can predict future change in BMO-MRW global thickness. The TS and NI locations found to be significant with all PERG parameters coincide with the earliest regions to show glaucomatous abnormalities, suggesting early functional loss can parallel BMO-MRW morphologic change. As RGCs become more dysfunctional, the BMO-MRW may degenerate, especially in the TS and NI areas. When it comes to the relationship of RNFL sectors with PERG parameters, localized RNFL defects have not been shown to be pathognomonic in glaucoma but are most often found in TI sector followed by TS sector, correlating with the rim configuration [2]. Mag and MagD were significantly correlated with global RNFL thickness and the NI sector (Table 7). Decreased RGCs would suggest not only reductions in rim area but also reductions in RNFL thickness, which supports our results (Table 7).

In the present study, we used rim area (mm^2^) as a global measure for NRR by means of Zeiss Cirrus OCT device, while SPECTRALIS® OCT and GMPE software provided sectorial measurements of the NRR in forms of BMO-MRW thicknesses. After controlling for disc area, significant relationships were found between all BMO-MRW sectors and rim area (Table 8). These findings suggest that, along with a global deterioration of the NRR and its significant thinning in PPG, there is a sectorial morphological change in the NRR in form of reductions in the BMO-MRW thickness measurements.

Furthermore, we found a significant association between RNFL thickness sectors and rim area in the I, NI, and NS sectors (Table 9). These findings agree with the pattern of glaucoma degeneration which has been shown to affect the inferior segment of the optic disc more often [62]. Localized RNFL defects have also been shown to be found most often in the temporal inferior sector followed by the temporal superior sector [2]. The RNFL damage seen in this study parallels the morphological changes occurring in the ONH. In Zangalli et al., BMO-MRW and RNFL thickness were assessed in healthy Brazilian individuals, with no significant association found [63]. The correlation between BMO-MRW and RNFL thickness in differently sized disc groups has been studied before; a small disc with a thick BMO-MRW and weak correlation with RNFL thickness may be falsely interpreted as normal or nonglaucomatous due to a normal BMO-MRW despite abnormal RNFL thickness [16]. However, similar to Zangalli et al.'s study, we did not find any significant correlation between BMO-MRW and RNFL thickness after controlling for disc area.

This study had many advantages. First, every participant had all his/her tests completed within the same day. Additionally, sufficient time was given to participants to recover between tests. Second, we used two SD-OCT devices to generate NRR data, where the rim area was used as a global measure of NRR, while BMO-MRW provided us with sectorial measurements by means of different software and algorithms. Third, we used the ss-PERG over ts-PERG, and it has been demonstrated that, in the latter, both P50 and N95 signals interact with signals from adjacent cells and neuronal generators, which complicate the interpretation of results. The ss-PERG modality, on the other hand, was less ambiguous. It increases the metabolic demand within the RGCs and leads to functional habituation [41]. Mag and MagD represent an objective indicator of RGC dysfunction and because these two parameters are essentially uncoupled, they reflect distinct aspects of RGC activity.

Limitations of our study include a relatively small sample size. More longitudinal studies are needed to investigate the relationships between PERG and ONH morphology after adjusting for disc size and including lamina cribrosa imaging that could shed some light on the mechanism of axonal damage. More OCT-angiography studies are needed to better understand the vascular abnormalities around the macula and the ONH and the effects of disc size on the capillary density and flow measures.

## 5. Conclusion

In this study, we report significant associations between PERG parameters and ONH morphology measurements such as rim area and BMO-MRW sectors. After controlling for disc size in our analysis, this relationship became even more significant despite using modern OCT algorithms that incorporated the confounding effects of disc size. Furthermore, Mag and MagD were strong predictors in rim area and BMO-MRW thickness variances. The ss-PERG provides objective, functional, quantitative, and qualitative information about RGC function. When examining PPG patients, we recommend use of ss-PERG with OCT derived ONH morphology measures while controlling for disc area to increase diagnostic accuracy of the devices and to circumvent underestimation or overestimation of the NRR.

## Figures and Tables

**Table 1:** Study characteristics.

*N* = 32 eyes (20 patients)	

	Mean ± SD
Age (years)	58.57 ± 14.60
Sex (% females)	13 females (65.00%)
IOP (mmHg)	17.73 ± 4.79
CCT (*µ*m)	549.50 ± 29.34
SE (D)	−0.50 ± 2.26

*Humphrey Visual Field*	
24-2 MD (dB)	0.25 ± 0.94
24-2 PSD (dB)	1.50 ± 0.33
24-2 VFI (%)	99.27 ± 0.91

*ss-PERG*	
Magnitude (*µ*V)	1.68 ± 0.61
MagnitudeD (*µ*V)	1.39 ± 0.64
MagnitudeD/Magnitude ratio	0.81 ± 0.14

IOP—intraocular pressure, CCT—central corneal thickness, SE—spherical equivalent, MD—mean deviation, PSD—pattern standard deviation, VFI—visual field index, ss-PERG—steady state pattern electroretinogram.

**Table 2:** OCT measurements.

*N* = 32 eyes (20 patients)	

	Mean ± SD
Rim area (mm^2^)	1.12 ± 0.18
Disc area (mm^2^)	1.89 ± 0.42
Average RNFL thickness (*µ*m)	90.5 ± 10.10

Average C/D ratio	0.64 ± 0.16
Vertical C/D ratio	0.62 ± 0.15
Cup volume (mm^3^)	0.32 ± 0.24

BMO-MRW global (*µ*m)	264.82 ± 50.52
BMO-MRW temporal (*µ*m)	189.64 ± 40.40
BMO-MRW temporal superior (*µ*m)	258.68 ± 53.59
BMO-MRW temporal inferior (*µ*m)	278.29 ± 59.16
BMO-MRW nasal (*µ*m)	290.75 ± 72.26
BMO-MRW nasal superior (*µ*m)	291.36 ± 60.18
BMO-MRW nasal inferior (*µ*m)	328.54 ± 64.96

RNFL global (*µ*m)	95.21 ± 10.23
RNFL temporal (*µ*m)	73.93 ± 17.07
RNFL temporal superior (*µ*m)	125.50 ± 25.05
RNFL temporal inferior (*µ*m)	147.11 ± 18.37
RNFL nasal (*µ*m)	73.54 ± 10.77
RNFL nasal superior (*µ*m)	97.43 ± 21.60
RNFL nasal inferior (*µ*m)	109.29 ± 23.65
RNFL superior (*µ*m)	110.18 ± 18.77
RNFL inferior (*µ*m)	123.36 ± 14.36

OCT—optical coherence tomography, RNFL—retinal nerve fiber layer, C/D—cup to disc, BMO-MRW—Bruch's membrane opening-minimum rim width.

**Table 3:** Partial correlation between pattern electroretinogram parameters and optic nerve head measurements, controlling for disc area.

	Rim area		Average C/D ratio		Vertical C/D ratio		Cup volume	
	*r*	*p* value	*r*	*p*value	*r*	*p* value	*r*	*p* value
Magnitude	0.518	0.003^*∗∗*^	0.072	0.702	−0.127	0.497	−0.322	0.078
MagnitudeD	0.503	0.004^*∗∗*^	0.034	0.857	−0.087	0.641	−0.239	0.195
MagnitudeD/Magnitude ratio	0.286	0.119	−0.124	0.505	0.015	0.935	0.091	0.626

^*∗∗*^*p* < 0.01; ^*∗*^*p* < 0.05.

**Table 4:** Associations of Magnitude and MagnitudeD with rim area (mm^2^), controlling for disc area (mm^2^).

	Step 1 (disc area)		Step 2 (Magnitude)			
	Δ*R*^2^	B (95% CI)	Δ*R*^2^	B (95% CI)	*R* ^2^	SE
Rim area (mm^2^)	0.002	0.021 (−0.137, 0.179)	0.268^a^	0.174 (0.065, 0.283)	0.270^a^	0.053

	Step 1 (disc area)		Step 2 (MagnitudeD)			
	Δ*R*^2^	B (95% CI)	Δ*R*^2^	B (95% CI)	*R* ^2^	SE
Rim area (mm^2^)	0.002	0.021 (−0.137, 0.179)	0.252^a^	0.160 (0.056, 0.265)	0.255^a^	0.051

Steps of the regression are shown separated by the columns. Δ*R*^2^ is the change in *R*^2^, B (95% CI) is the B coefficient and 95% confidence interval ranges, *R*^2^ for total *R*^2^ of the model, and SE is the standard error of the estimate of the final model. ^*a*^*p* < 0.01.

**Table 5:** Partial correlation between pattern electroretinogram parameters and Bruch's membrane opening-minimum rim width, controlling for disc area.

	Global		Nasal		Nasal inferior		Nasal superior		Temporal		Temporal inferior		Temporal superior	
	*r*	*p* value	*r*	*p* value	*r*	*p* value	*r*	*p* value	*r*	*p* value	*r*	*p* value	*r*	*p* value
Magnitude	0.374	0.055	0.214	0.284	0.508	0.007^*∗∗*^	0.294	0.136	0.431	0.025^*∗*^	0.198	0.321	0.400	0.039^*∗*^
MagnitudeD	0.416	0.031^*∗*^	0.260	0.191	0.545	0.003^*∗∗*^	0.366	0.060	0.411	0.033^*∗*^	0.247	0.214	0.433	0.024^*∗*^
MagnitudeD/Magnitude ratio	0.407	0.035	0.335	0.088	0.431	0.025^*∗*^	0.459	0.016^*∗*^	0.225	0.259	0.269	0.174	0.408	0.034^*∗*^

^*∗∗*^*p* < 0.01; ^*∗*^*p* < 0.05.

**Table 6:** Associations of MagnitudeD and MagnitudeD/Magnitude ratio with Bruch's membrane opening-minimum rim width global (mm^2^), controlling for disc area (mm^2^).

	Step 1 (disc area)		Step 2 (MagnitudeD)			
	Δ*R*^2^	B (95% CI)	Δ*R*^2^	B (95% CI)	*R* ^2^	SE

BMO-MRW global (mm^2^)	0.228^a^	−57.979 (−101.047, −14.911)	0.134^b^	38.921 (3.872, 73.970)	0.361^b^	17.018

	Step 1 (disc area)		Step 2 (MagnitudeD/Magnitude ratio)			
	Δ*R*^2^	B (95% CI)	Δ*R*^2^	B (95% CI)	*R* ^2^	SE

BMO-MRW global (mm^2^)	0.228^a^	-57.979 (−101.047, −14.911)	0.128^b^	129.024 (9.589, 248.460)	0.355^b^	57.991

Steps of the regression are shown separated by the columns. Δ*R*^2^ is the change in *R*^2^, B (95% CI) is the B coefficient and 95% confidence interval ranges, *R*^2^ for total *R*^2^ of the model, and SE is the standard error of the estimate of the final model. BMO-MRW—Bruch's membrane opening-minimum rim width. ^*a*^*p* < 0.01. ^*b*^*p* < 0.05.

**Table 7:** Partial correlation analysis between pattern electroretinogram parameters and retinal nerve fiber layer sectors, controlling for disc area.

	Global		Inferior		Superior		Nasal		Nasal inferior		Nasal superior		Temporal		Temporal inferior		Temporal superior	
	*r*	*p* value	*r*	*p* value	*r*	*p* value	*r*	*p* value	*r*	*p* value	*r*	*p* value	*r*	*p* value	*r*	*p* value	*r*	*p* value
Magnitude	0.444	0.020^*∗*^	0.534	0.004^*∗∗*^	0.516	0.006^*∗∗*^	0.282	0.155	0.422	0.028^*∗*^	0.490	0.009^*∗∗*^	−0.112	0.577	0.207	0.300	0.330	0.093
MagnitudeD	0.449	0.019^*∗*^	0.583	0.001^*∗∗*^	0.514	0.006^*∗∗*^	0.336	0.086	0.424	0.028^*∗*^	0.507	0.007^*∗∗*^	−0.159	0.428	0.294	0.137	0.332	0.091
MagnitudeD/Magnitude ratio	0.334	0.089	0.509	0.007^*∗∗*^	0.428	0.026^*∗*^	0.422	0.028^*∗*^	0.307	0.119	0.513	0.006^*∗∗*^	−0.306	0.121	0.335	0.088	0.223	0.264

^*∗∗*^*p* < 0.01; ^*∗*^*p* < 0.05.

**Table 8:** Partial correlation between Bruch's membrane opening-minimum rim width and rim area, controlling for disc area.

	Global		Temporal		Temporal superior		Temporal inferior		Nasal		Nasal superior		Nasal inferior	
	*r*	*p* value	*r*	*p* value	*r*	*p* value	*r*	*p* value	*r*	*p* value	*r*	*p* value	*r*	*p* value
Rim area	0.726	0.001^*∗∗*^	0.558	0.002^*∗∗*^	0.672	0.001^*∗∗*^	0.497	0.008^*∗∗*^	0.579	0.002^*∗∗*^	0.766	0.001^*∗∗*^	0.673	0.001^*∗∗*^

^*∗∗*^*p* < 0.01; ^*∗*^*p* < 0.05.

**Table 9:** Partial correlation between retinal nerve fiber layer sectors and rim area, controlling for disc area.

	Global		Inferior		Superior		Nasal		Nasal inferior		Nasal superior		Temporal		Temporal inferior		Temporal superior	
	*r*	*p* value	*r*	*p* value	*r*	*p* value	*r*	*p* value	*r*	*p* value	*r*	*p* value	*r*	*p* value	*r*	*p* value	*r*	*p* value
Rim area	0.112	0.578	0.389	0.045^*∗*^	0.330	0.092	0.217	0.277	0.422	0.028^*∗*^	0.620	0.001^*∗∗*^	−0.496	0.008^*∗∗*^	0.064	0.753	−0.024	0.907

^*∗∗*^*p* < 0.01; ^*∗*^*p* < 0.05.

## Data Availability

The data that support the findings of this study are available upon request from the corresponding author, AT. The data are not publicly available due to the fact that they contain information that could compromise the privacy of research participants.
